# Expression of Long Non-Coding RNA *H19* in Acute Lymphoblastic Leukemia

**DOI:** 10.22074/cellj.2022.8315

**Published:** 2022-12-28

**Authors:** Marjan Asadi, Mohammad Ali Gholampour, Farzad Kompani, Shaban Alizadeh

**Affiliations:** 1. Hematology Department, School of Allied Medicine, Tehran University of Medical Science, Tehran, Iran; 2. Hematology Department, Faculty of Medical Sciences, Tarbiat Modares University, Tehran, Iran; 3. Division of Hematology and Oncology, Children's Medical Center, Pediatrics Center of Excellence, Tehran University of Medical Science, Tehran, Iran

**Keywords:** Acute Lymphoblastic Leukemia, lncRNA, H19, Hypoxia

## Abstract

**Objective:**

Long non-coding RNA (lncRNA) H19 has essential roles in growth, migration, invasion, and metastasis of most cancers. H19 dysregulation is present in a large number of solid tumors and leukemia. However, the expression level of H19 in acute lymphoblastic leukemia (ALL) has not been elucidated yet. The current study aimed to explore *H19* expression in ALL patients and cell lines.

**Materials and Methods:**

This experimental study was conducted in bone marrow (BM) samples collected from 25 patients with newly diagnosed ALL. In addition, we cultured the RPMI-8402, Jurkat, Ramos, and Daudi cell lines and assessed the effects of internal (hypoxia) and external (chemotherapy medications L-asparaginase [ASP] and vincristine [VCR]) factors on *h19* expression. The expressions of *H19, P53, c-Myc, HIF-1α* and *β-actin* were performed using quantitative real-time polymerase chain reaction (qRT-PCR) method.

**Results:**

There was significantly increased *H19* expression in the B-cell ALL (B-ALL, P<0.05), T-cell ALL (T-ALL, P<0.01) patients and the cell lines. This upregulation was governed by the *P53, HIF-1α,* and *c-Myc* transcription factors. We observed that increased *c-Myc* expression induced *H19* expression; however, P53 adversely affected *H19* expression. In addition, the results indicated that chemotherapy changed the gene expression pattern. There was a considerable decrease in *H19* expression after exposure to chemotherapy medications; nonetheless, hypoxia induced *H19* expression through *P53* downregulation.

**Conclusion:**

Our findings suggest that H19 may have an important role in pathogenesis in ALL and may act as a promising and potential therapeutic target.

## Introduction

Acute lymphoblastic leukemia (ALL) is the most prevalent leukemia in childhood. It is characterized by overproduction and accumulation of lymphoblasts in the bone marrow (BM) (1). In addition, ALL relapse is the fundamental cause of treatment failure in 15-20% of patients (2, 3). Therefore, the exploration of novel functional molecules that play a role in ALL pathogenesis could be effective therapeutic targets for this disease.

Long non-coding RNAs (lncRNAs) are non-proteincoding transcripts longer than 200 nucleotides. The results from recent studies have revealed various functions of lncRNAs in molecular mechanisms of biological and pathological processes (4-7). H19 was the first lncRNA to be discovered and submitted for genomic imprinting (8-10). H19 has a role in embryogenesis and tumorigenesis (11). H19 acts as a molecular sponge for miRNA-138 and miRNA200a, the precursors of microRNA675 , which interact with epigenetic polycomb proteins. H19 has an indispensable role in enhancing cell proliferation, differentiation, migration, invasion, and chemoresistance (12, 13). Initially, H19 was reported to be a tumor suppressor (14, 15), however, recent evidence has shown that H19, as an oncogene, is overexpressed in breast (16-18), liver (18), endometrial (19), lung, cervical, and esophageal cancers (5). A similar pattern of H19 expression is observed in various types of leukemia, including chronic myeloid leukemia (CML) (20, 21) and acute myeloid leukemia (AML) (22), as well. These observations together suggest dual roles for H19 as an oncogene or a tumor suppressor in various cancers (20). Therefore, future studies could evaluate the potential for lncRNAs as therapeutic targets or prognostic biomarkers in cancer treatments (23).

According to previous studies, some transcriptional factors can regulate and change the molecular functions of H19. For instance, c-Myc is a transcriptional factor that plays an oncogenic role through attaching to conserved E-boxed in the vicinity of the imprinting control region (ICR), to induce acetylation of histones and the H19 promoter. c-Myc induces H19 expression and may contribute to tumor etiology or function as an oncogene (12, 24, 25). c-Myc was first identified in Burkitt’s lymphoma. Its activation is caused by a chromosomal translocation. However, deregulation of c-Myc expression has been observed along with poor patient survival in numerous human cancers, including carcinomas of the prostate, breast, lung, as well as leukemia, and lymphoma (26). Conversely, H19 and p53 mutually counter-regulate each other (27). The p53 tumor suppressor is a transcriptional factor that regulates various anti-proliferative processes and downregulates H19 expression by inducing DNA demethylation at the ICR of H19 (28-30). Various physiological processes such as cell metabolism, survival, proliferation, and angiogenesis play crucial roles in pathological scenarios (31, 32). A hypoxic region is present in ALL BM, and it is considered to be a determinative factor for both a therapeutic response and tumor progression. A complicated cellular network of genes is involved in tumor progression (33). Researchers have reported that H19 is a part of this network because its expression could be upregulated in hypoxic stress (34, 35). *HIF-1α* is a crucial factor responsible for H19 induction under hypoxic conditions and acts as a p53 inhibitor, leading to tumor growth. Hypoxia can indirectly upregulate H19 expression by inhibition of p53 (24, 35). 

H19 is overexpressed in various cancerous tissues and has been shown to be associated with carcinogenesis, cell proliferation and differentiation, metastasis, poor prognosis, and tumor growth in various cancers, while normal expression of H19 has been shown in healthy individuals. This LncRNA may act as a novel diagnostic and prognostic marker for malignancies. 

## Materials and Methods

### Cell lines and patient samples

In this experimental study, we cultured the RPMI-8402, Jurkat, Ramos, and Daudi cell lines inRPMI-1640 (Gibco Life Technique, Germany) with 10 % heat-inactivated fetal bovine serum (FBS, Sigma-Aldrich, USA), 100 U/ml penicillin, and 100 µg/ml streptomycin. The cells were maintained in a humidified incubator with 5% CO_2_ at 37°C. We purchased cell lines from National Cell Bank of Iran (NCBI, Pasteur Institute of Iran, Tehran, Iran). The patient samples were collected from BM of 25 newly diagnosed ALL individuals, 15 with B-ALL and 10 with T-ALL, and 20 healthy donors admitting to the Children’s Medical Center, Tehran, Iran. The medical Ethics Committee of Tehran University (IR.TUMS. REC.1394.2201) permitted this study. 

### B and T cell isolation

BM from both healthy donors and patients were collected in EDTA tubes and mononuclear cells of BM (BM-MNCs) were isolated through Ficoll-Hypaque (Lymphodex, Germany). The isolated cells were magnetically labeled with Anti- CD3 and CD19 MACS MicroBeads (Miltenyi Biotech, Germany). Next, the samples were washed using 1-2 mL of phosphate-buffered saline (PBS) buffer per 10^6^ cells and subsequently centrifuged for 10 minutes at 300x g. The cell suspension was placed onto the separation column and the flow-through that contained the unlabeled cell was collected. The column was then removed and the retained cells were washed, collected and considered as a positively selected cell fraction. The isolated cells from healthy individuals were considered to be the control group for evaluation of gene expression in patients with ALL. 

### Flow cytometry analysis

Flow cytometry analysis was conducted to assess the purity of the isolated B and T cells using CD19-FITC and CD3-PE-conjugated antibodies, respectively (ebioscience, Thermo Fisher Science, USA). The antibody binding step was performed for 45 minutes at 4°C in the dark. Afterward, the cells were resuspended in 100 µl of 0.5% PBS. The analysis was carried out by a flow cytometer (FACScalibur, Becton Dickenson, MA, USA).

### RNA extraction and quantitative real-time polymerase chain reaction

Total RNA was extracted using RNeasy Mini Kit (Qiagen, Hilden, Germany) according to manufacturer’s instruction. Next, using the revert Aid First-Strand cDNA Kit (Fisher Scientifiec, MA, USA) protocol cDNA was synthesized. Applied Biosystem 7300 real-time PCR System (Applied Biosystem, Foster City, USA) was used for quantitative real-time polymerase chain reaction (qRT-PCR). The following primers were used for qPCR: 

*H19* sense: 5ˊ-TGTTTCTTTACTTCCTCCACGG-3ˊ 

antisense: 5ˊ- TTCCTCTAGCTTCACCTTCCAG-3ˊ

*c-Myc* sense: 5ˊ-TTCGGGTAGTGGAAAACCAG-3ˊ

antisense: 5ˊ-AGTAGAAATACGGCTGCACC-3ˊ

*P53* sense: 5ˊ- TCAACAAGATGTTTTGCCAACTG-3ˊ

antisense: 5ˊ- ATGTGCTGTGACTGCTTGTAGATG-3ˊ

HIF1-α sense: 5ˊ- TTCACCTGAGCCTAATAGTCC-3ˊ

antisense: 5ˊ- CAAGTCTAAATCTGTGTCCTG-3ˊ

*β-actin* sense: 5ˊ- TGAAGATCAAGATCATTGCTCCTC-3ˊ 

antisense: 5ˊ- AGTCATAGTCCGCCTAGAAGC-3ˊ

*β-actin* was used for normalization. Relative gene expression was calculated based on 2^-ΔΔCT^ method. All experiments had three technical replicates.

### Hypoxia treatment

ALL cell lines were cultured for 24 hours at 37°C in an anaerobic incubator (Ruskin Technologies, Pencoed, Wales, UK) with constant hypoxic environment (2% O_2_, 93% N_2_, and 5% CO_2_) and 90% humidity. The time of inducing hypoxic condition was selected based on previous studies (36). The cell lines cultured under Normoxia conditions were incubated in 5% CO_2_ at 37°C.

### Treatment with chemotherapy drugs and dose selection

The effects of the chemotherapy drugs L-asparaginase (ASP, Paronal, Christiaens, The Netherlands) and vincristine (VCR, TEVA Pharma, Utrecht, The Netherlands) were assessed on *h19, c-myc, p53*, and *hif-1α* expressions in all of the cell lines. The logarithmic ranges of drugs were selected based on previous studies (2, 3). We used the 25-200 ng/mL doses of ASP and 2-16 ng/mL doses of VCR in our experiments to determine the lethal concentration of 50% (LC50%). Next, we assessed the target gene expressions in the ALL cell lines.

### MTT assay

Cell viability was performed by dimethylthiazol diphenyl tetrazolium bromide (MTT, Sigma, Chemical, St Louis, MO, USA). ALL cell lines were treated with VCR (25-200 ng/mL) and ASP (2-16 ng/mL) as mentioned above. Untreated cell lines were cultured as the control groups. After 48 hours, a standard concentration of MTT (10 µM) was added to every well and then 100 µM of dimethyl sulfoxide (DMSO) was added to dissolve the formazan. The optical density was evaluated using a microplate reader (Anthos2020, Salzburg, Austria) at 570 nm.

### Annexin V-fluorescein staining assay

After treatment with the chemotherapy drugs, we used BioscienceTM Annexin V-FITC Apoptosis in the ALL cell lines. Briefly, the cells were washed with phosphatebuffered saline (PBS) and stained with propidium iodide (PI) and Annexin V (FITC) for 20 minutes at room temperature. The samples were analyzed by a flow cytometer (FACScalibur, Becton Dickenson, MA, USA) with 10000 events acquired for each sample.

### Statistical analysis

All data are presented as standard deviation (SD). We used Mann-Whitney U test to analyze two independent samples of patients’ data. A Mann-Whitney test is used when we have a continuous level variable measured for all observations in two groups and we want to test if the distribution of this variable is different in the two groups but we are unable to assume normality in both groups. Statistical analysis were done with student’s test for differences in each two-group comparison and one-way ANOVA was used to determine the differences among at least three groups. GraphPad Prism, version 6.07 (GraphPad Software, USA) was used for statistical analysis. A P<0.05 was defined as statistically significant.

## Results

### *H19 *expression was upregulated in T and B lymphocytes from ALL patients

**Fig 1:**
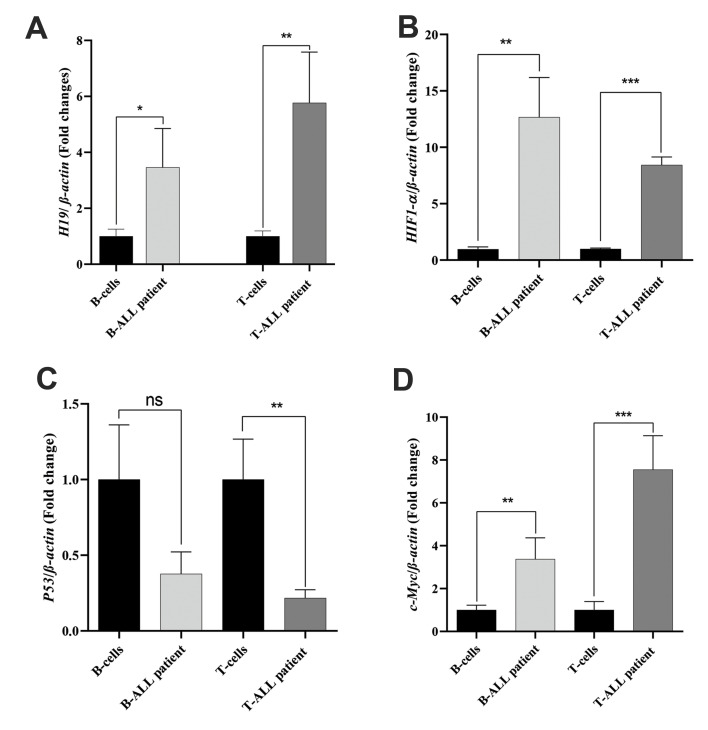
Gene expression in patients with ALL. All gene expressions were detected by quantitative real time PCR. T and B cells were extracted from B-ALL (n=15) and T-ALL (n=10) patients and compared with healthy donors (n=20) as the control group. **A. ***H19* expression in ALL patients and control group. **B. ***HIF-1α*, **C. ***P53*, and **D. ***c-Myc* expression levels in patient samples. Representative experiments performed in triplicate. The graphs demonstrate the standard deviation (SD). ALL; Acute lymphoblastic leukemia, B-ALL; B-cell acute lymphoblastic leukemia, T-ALL; T-cell acute lymphoblastic leukemia, PCR; Polymerase chain reaction, ns; Not significant, *; P<0.05, **; P<0.01, and ***; P<0.001.

In this study 25 patients were enrolled. In experimental group, there were 17 males (68%) and 8 females (32%). In the control group, there were 11 males (55%) and 9 females (45%). The *H19* expression levels in B-ALL and T-ALL cases were 2.51 (1.81 to 4.12) and 4.72 (2.71 to 9.48), respectively (Fig .1A). Our findings showed that *H19* expression was higher in the experimental B-ALL (P<0.05) and T-ALL (P<0.01) samples in comparison to the control group. The BM microenvironment in ALL is hypoxic; a condition that controls the expressions of other genes, particularly *H19*. Therefore, we assessed *HIF-1α* expression in patients and healthy samples. The results showed a significantly high *HIF-α* expression in B-ALL (12 folds, P<0.01) and T-ALL (8 folds, P<0.001) patients compared to the control group (Fig .1B). Because *H19* and *c-Myc*, as well as *P53*, are frequently co-amplified in cancer, we examined the expression levels of *c-Myc* and *P53* in the ALL patients and healthy subjects. *P53* expression was downregulated in both B-ALL (0.4-fold, ns) and T-ALL (0.3-fold, P<0.01) patients along with upregulation of *c-Myc* in B-ALL (3-fold, P<0.01) and T-ALL (7-fold, P<0.001) patients (Fig .1C, D). In addition, as seen in Supplementary section, flow cytometry analysis of isolated T and B lymphocytes showed that T cells expressed CD3 (98.3%) and B cells expressed CD19 (88.7%) as control groups. Clinical sample characteristics are summarized in Table 1.

**Table 1:** The demographic and clinicopathologic characteristics of newly diagnosed ALL patients (25 patients)


Clinical factor	Numbers/Percentage

Male	17 (68.0)
Female	8 (32.0)
Median age (Y)	28 (6-30)
BM blast	81 (20-95)
Median WBC (10^6^/μl)	19000 (700-400000)
Immunophenotype B-ALL	15 (60.0)
CD19+median	69.8
CD20+median	11.2
CD22+median	42.8
Cyto CD 79α	63.8
CD34+median	42.8
TdT+median	51.5
Immunophenotype T-ALL	10 (40.0)
CD2+median	96.1
CD3+median	75.9
CD5+median	85.1
CD7+median	88.5
CD34+median	60.4
TdT+median	53.0


Data are presented as n (%) or %. BM; Bone Marrow, WBC; White blood cell, B-ALL; B-cell acute lymphoblastic leukemia, and T-ALL; T-cell acute lymphoblastic leukemia.

### *H19* expression increased in ALL cell lines

**Fig 2:**
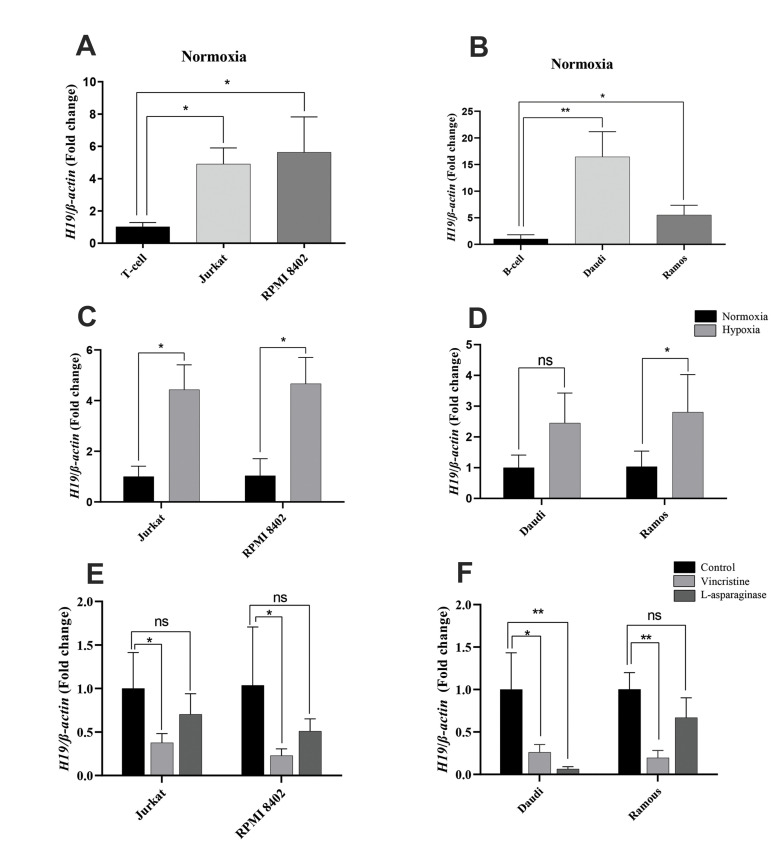
*H19* expression in ALL cell lines. Gene expression was assessed by q-RT PCR. **A, B. ***H19* expression was analyzed in both B and T ALL cell lines and compared to normal B and T cells, respectively. **C, D. **Analysis of *H19* expression in cell lines after 24 hours of exposure to hypoxic stress. **E, F. **Expression of *H19* was evaluated after treatment with VCR and ASP. The data are from three independent experiments, each done in triplicate. ALL; Acute lymphoblastic leukemia, q-RT PCR; Quantitative real-time polymerase chain reaction, VCR; Vincristine, ASP; L-asparaginase, ns; Not significant, *; P<0.05, and **; P<0.01.

We conducted an *in vitro* examination of *H19* in the B and T lymphocyte-derived cell lines. The *H19* expression levels in Jurkat and RPMI-8402 cells showed an almost equivalent increase in *H19* expression (4.8 folds, P<0.05, Fig .2A). Expression of *H19* in the B cell lines of Daudi and Ramos were 16 folds, P<0.01 and 6 folds, P<0.05, respectively (Fig .2B). This finding suggested that cell lines had higher *H19* expression compared with the B and T lymphocytes. We also evaluated *H19* expression under hypoxic conditions after 24 hours. Interestingly, we found elevated expression levels of *H19* in the T cell lines (about 4-folds, P<0.05) and B cell lines (at least 2-folds, Daudi ns, Ramous P<0.05, Fig .2C, D). To further assess the molecular mechanism of *H19* in ALL pathogenesis, we treated the cell lines with VCR and ASP drugs. Expression of *H19* decreased by (0.24-fold, P<0.05) and (0.4-fold, P<0.05) in the Jurkat and RPMI-8402 cells, and (0.22- fold, P<0.05) and (0.29-fold, P<0.01) in the Daudi and Ramous cells, respectively. Treatment with ASP caused an insignificant downregulation of *H19* expression in the ALL cell lines, with the exception of the Daudi cells (Fig .2E, F). These findings demonstrated that *H19* expression was declined in treated cell lines in comparison to untreated cells.

### Evaluation of cell line viability after culture in the presence of chemotherapy drugs

**Fig 3:**
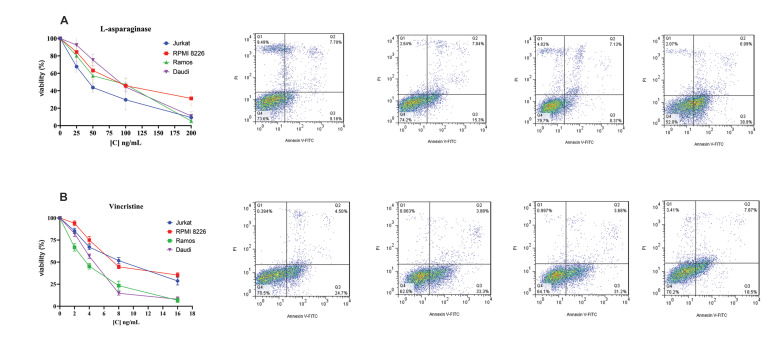
The effects of various doses of VCR and ASP on viability of ALL cell lines. **A.** MTT test was performed to investigate the survival of ALL cell lines at 48 hours after treatment with VCR and ASP. **B.** Staining with Annexin-V and PI assessed apoptosis. The histogram demonstrates the ratio of apoptotic or necrotic cells. Annexin V-/PI-, Annexin V+/PI-, Annexin V+, and PI+ represented live cells, early apoptotic cells, late apoptotic cells, necrotic cells, respectively. The data are from two independent experiments, each done in duplicate. VCR; Vincristine, ASP; L-asparaginase, ALL; Acute lymphoblastic leukemia, MTT; Dimethylthiazol diphenyl tetrazolium bromide, VCR; Vincristine, ASP; L-asparaginase, and PI; Propidium iodide.

We used the MTT method to evaluate the viability of the cell lines with different doses and concentrations of VCR and ASP for 48 hours. The mean viability among the control and the treated groups was significant and is shown in Supplements. LC50 values were evaluated at the specific drug concentrations used for the cytotoxicity assay. To determine the effects of VCR and ASP on apoptosis of the ALL cell lines, we examined apoptotic cell death through AnnexinV and PI staining. The results showed that treatment with VCR and ASP induced apoptosis, as apoptosis was higher in the treated groups compared to the untreated cells (Fig .3).

**Fig 4:**
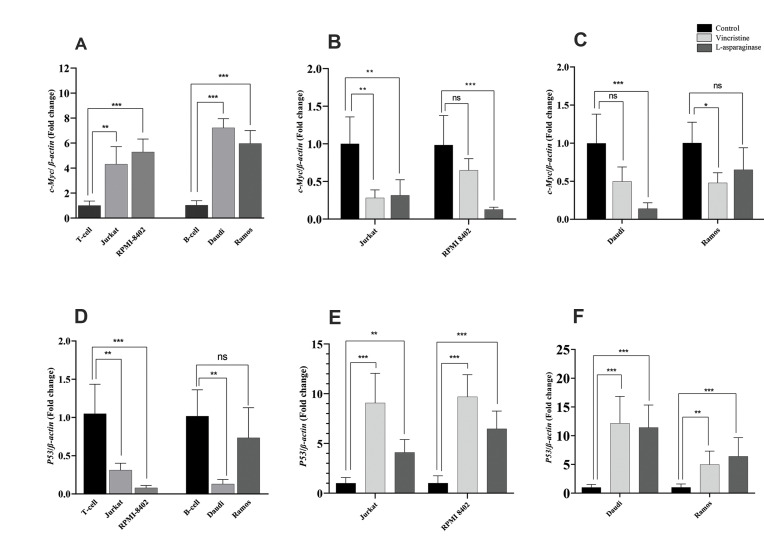
Expression of *H19*-related genes in the ALL cell lines. Gene expression was carried out through quantitative real-time PCR and the treated cell lines were compared with untreated controls. **A-C. **Expression of *c-Myc* was evaluated in B- and T- ALL cell lines and its relative expression was measured after a 48 hours of treatment with VIN and ASP. **D-F.** The relative expression levels of *P53* was measured in ALL cell lines and treated ones and compared to control group. The data are cultures from three independent experiments, each done in duplicate. ALL; Acute lymphoblastic leukemia, PCR; Polymerase chain reaction, VCR; Vincristine, ASP; L-asparaginase, ns; Not significant, *; P<0.05, **; P<0.01, and ***; P<0.001.

### Expressions of related genes in the ALL cell lines

**Fig 5:**
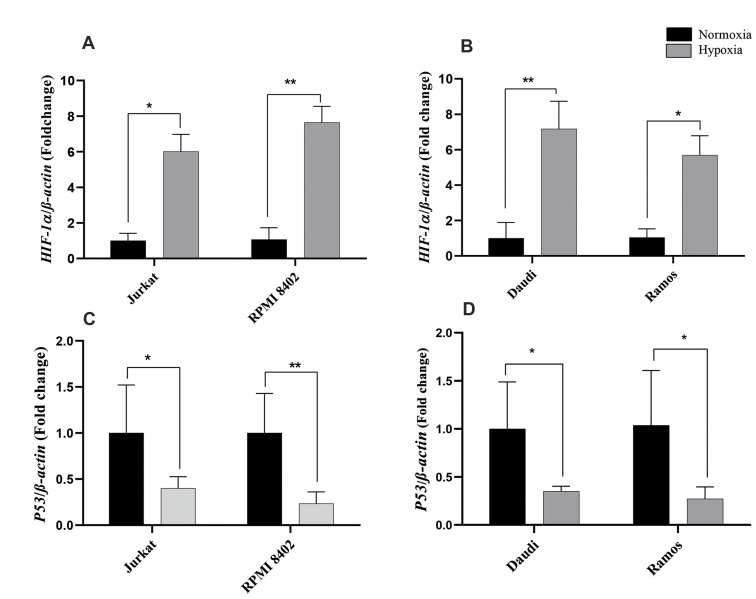
Expression of *c-Myc* and *P53* genes in the ALL cell lines after hypoxic condition. **A, B. **Expression of HIF-1a was evaluated by qRT-PCR after the cell lines were put into hypoxic condition for 24 hours. **C, D.** Expression of *P53* was evaluated after exposure to the hypoxic condition in comparison to normoxic culture conditions. The data are cultures from three independent experiments, each done in triplicate. ALL; Acute lymphoblastic leukemia, qRTPCR; Quantitative real time polymerase chain reaction, *; P<0.05, and **; P<0.00.

*H19* expression is related to various genes, particularly *HIF-1α, P53*, and *c-Myc*; therefore, we evaluated the expression levels of these genes in ALL cell lines. The relative expression of *c-myc* was significantly elevated in the T-ALL cell lines compared with the T-lymphocytes (about 4-folds, P<0.01 (Jurkat), P<0.001 (RPMI8402), and in the B-ALL cell lines compared with the B-lymphocytes (about 7-folds, P<0.001, Fig .4A). After treatment with VCR or ASP, *c-Myc* expression was significantly decreased in all cell lines; it was almost the same in Jurkat and Ramos cells after treatment. However, RPMI-8402 and Daudi cells had more robust decreases in *c-Myc* expression after treatment with ASP compared to VCR (Fig .4B, C). In contrast to the *c-Myc* expression pattern, there was a significant downregulation of *P53* expression in all of the cell lines, with the exception of Ramos cells (Fig .4D). On the other hand, *P53* expression was upregulated remarkably in all cell lines treated with either ASP or VCR. *P53* expression was significantly higher in the VCR-treated groups compared to the ASPtreated Jurkat cells (9-folds, P<0.001 vs. 4-folds, P<0.01) and RPMI-8402 cells (10-folds vs. 6-folds, P<0.001), respectively. B cell lines had approximately the same expression pattern of *P53* in the treated groups. Its expression was about 11.8 folds, P<0.001 in Daudi cells and about 5.7 folds P<0.01 in Ramous cells after treatment with VCR and ASP (Fig .4E, F). This finding showed that chemotherapy treatments upregulated *P53* expression, but downregulated *c-Myc*, which may ultimately lead to downregulation of *H19* expression. In addition, *H19* expression in hypoxic condition was related to HIF-1a gene through blocking *P53* expression; therefore, we evaluated the expression level of HIF-1a and *P53* in ALL cell lines. Expression of HIF-1a was upregulated in the B and T ALL cell lines (about 6-folds) (Fig .5A, B), whereas upregulation of *P53* was observed in all cell lines (Fig .5C, D). This result showed that upregulation of *H19* could be related to these genes in hypoxic condition.

## Discussion

Gene expression is involved in various physiological processes, including differentiation, apoptosis, cell proliferation, and metastasis, and is directly regulated by LncRNA. It has been suggested that these LncRNAs could acts as tumor suppressors or oncogenes that make them potential diagnostic and prognostic biomarker in cancers (12). The expression level of H19 and its presumptive role in ALL have not been entirely appreciated. In the present study, we found that the level of *H19* expression increases in ALL patients that might have a tumor promoter in ALL, which is in harmony with other studies. In previous studies, Zhang et al. (22) have reported that upregulation of *H19* expression in BM-MNCs from newly diagnosed AML patients; these changes were reported in eight cell lines derived from AML patients. In another study on hematologic malignancies, Guo et al. (20) observed higher expression of *H19* in cell lines with Bcr-Abl transformation and in primary cells from patients with chronic myelogenous leukemia (CML). Downregulation of the *H19* expression has been shown to sensitize leukemic cells to imatinib-induced apoptosis and inhibit tumor growth resulting from Bcr-Abl transformation. In addition to hematologic malignancies, other investigations also reported overexpression of *H19* in numerous solid cancers such as hepatic, bladder, gastric, lung, and ovarian cancers (5).

In our study, we evaluated *H19* expression in ALL patients. Interestingly, an analogous increase in *H19* expression occurred in both T-ALL and B-ALL patients as well as in the cell lines; however, *H19* expression was significantly higher in the cell lines. 

Previous studies verified the gene-gene interactions between *H19* and crucial genes related to survival and proliferation, such as *c-Myc* and *P53*. Thus, we further assessed *c-Myc* expression in ALL. *H19* and *c-Myc* expression levels were significantly increased in ALL patients and cell lines. We assumed that there could be a correlation between elevated expressions of these two genes. The Ramos and Jurkat cell lines had significantly higher *c-Myc* expression and significantly greater *H19* expression. The results of other studies showed that c-Myc could upregulate *H19* expression, Guo et al. (20) observed this direct induction in K562 cell lines. They found that knockdown of *c-myc* expression significantly decreased *H19* expression. The same relationship has been also reported in breast, esophageal, and colorectal tumors (21, 22).

In contrast to *c-Myc*, which has been demonstrated to increase H19 expression and enhance cell growth and tumorigenesis, P53 is the most important tumor suppressor gene in cancer that is negatively associated with H19 expression. p53 not only represses the promoter activity of the H19 gene, it also suppresses H19 expression at the epigenetic level (9). Dugimont et al. (28) reported the inhibitory effect of P53 on H19 expression by the luciferase assay in HeLa (cervical cancer) and Calu-6 (human pulmonary adenocarcinoma) cell lines. This was also confirmed in the AGS cell line (gastric cancer), where ectopic H19 expression increased cell proliferation, and H19 siRNA treatment was associated with P53 inactivation and cell apoptosis (37). Our findings demonstrated an inverse relationship between P53 and H19 expressions. We observed decreased *P53* expression in the ALL patients along with elevated *H19* expression. The same correlation occurred in the cell lines where higher expression of *H19* co-occurred with decreased *P53* expression. 

Apart from gene-gene interactions, environmental stimuli could effectively change the cellular process, and particularly affect cell growth and apoptosis, as well. Notably, previous studies have indicated that hypoxic stress induces H19 expression and could increase cell proliferation in malignancies. It has been confirmed that hypoxia regulates leukemia progression and causes resistance to radiotherapy and chemotherapy (33, 38). HIF-1α is a key regulator of the hypoxic response and a major oxygen homeostasis regulator. It is a crucial factor that is responsible for H19 RNA induction under hypoxic conditions by inhibiting P53 expression (39).

Matouk and colleagues demonstrated that hypoxia upregulates H19 expression via an inhibitory effect of HIF-1a on P53 expression in hepatocellular and bladder carcinoma (25). In another study, they manipulated different lineage sources of carcinoma and overexpressed H19. This modification was along with a decreased level of P53 expression. They demonstrated a tight connection between the P53 gene and H19 expression under hypoxic stress, which was determined by semi-quantitative RTPCR. These researchers also found that knockdown of HIF-1α remarkably diminished *h19* expression under hypoxic condition (35).

*H19* expression was significantly increased in ALL patient samples, which could be due to hypoxia-like conditions in BM. The ALL cell lines confirmed that the hypoxic condition and *HIF-1α* induction caused *P53* suppression and a simultaneous up-regulation of *H19*.

We examined the expression of *H19* and its related genes in B and T ALL patients and cell lines. Next, we examined the potential application of *H19* in therapeutic conditions by treating the ALL cell lines with two common chemotherapy drugs prescribed for ALL patients, VCR and ASP. Next, we evaluated the expressions of *H19* and its associated genes following drug treatments. Expression of *H19* in the treated leukemia cell lines was downregulated compared to the untreated cell lines. In addition, assessment of the related genes demonstrated that suppression of *H19* occurred along with significantly elevated *P53* and lower *c-Myc* expressions. This pattern could suggest the possible prognostic applications of *H19* in patients with ALL. Several studies also explored the role of H19 in the clinical status of cancer patients. Zhang et al. (22) reported that H19 overexpression correlated with poor overall survival and chemotherapy response among AML patients. However, H19 expression in patients with AML who achieved complete remission after induction therapy was lower compared to the patients with relapses. In another study, Guo et al. (20) found that tumor formation was attenuated by h19 knockdown in a mouse xenograft model. They observed that *H19* repression in K562 cell line could significantly inhibit tumor progression. Knocking down *H19* RNA by siRNA resulted in inhibiting tumorigenicity in hepatocellular cells (Hep3B) and human bladder carcinoma cells (UMUC3) *in vivo* (25).

## Conclusion

It has been demonstrated that H19 expression might act as a novel target for prognosis prediction as well as the means for assessing the clinicopathologic features in various cancers. Our findings show that the expression of *H19* increased in B and T ALL patients and cell lines, which may be related to the expression of *P53, c-Myc*, and *HIF-1α*. Interestingly, *H19* expression was significantly upregulated by hypoxic condition, while a decreased expression of this gene was observed after treatment with chemotherapy drugs. Although our study suggests that *H19* could be accounted for a potential therapeutic target or useful predictive biomarker in ALL patients, further investigation is needed to identify the molecular mechanisms underlying *H19* function in pathological process and/or carcinogenesis in this disease.
